# Supplementary material for: Estimating Rates of Progression and Predicting Future Visual Fields in Glaucoma Using a Deep Variational Autoencoder
Source: Sci Rep. 2019 Dec 2;9:18113. doi: 10.1038/s41598-019-54653-6 (PMC6888896; doi:10.1038/s41598-019-54653-6)
Supplement: Supplementary file 1 — Supplementary Information [file 41598_2019_54653_MOESM1_ESM.docx]

**Supplementary Information for the Manuscript:** “Estimating Rates of Progression and Predicting Future Visual Fields in Glaucoma Using a Deep Variational Autoencoder”

Samuel I. Berchuck;^1,2^ Sayan Mukherjee;^3^ Felipe A. Medeiros ^1^

1. Duke Eye Center and Department of Ophthalmology, Duke University, Durham, North Carolina.
2. Department of Statistical Science and Forge, Duke University, Durham, North Carolina.
3. Departments of Statistical Science, Mathematics, Computer Science, Biostatistics & Bioinformatics, Duke University, Durham, North Carolina.

**Correspondence:** Felipe A. Medeiros, MD, PhD, Visual Performance Laboratory, Duke Eye Center and Department of Ophthalmology, Duke University, 2310 Erwin Rd, Durham, NC, 27710, E-mail: [felipe.medeiros@duke.edu](mailto:felipe.medeiros@duke.edu).

**Supplementary Table S1:** Comparing the visual field progression hit rate of mean deviation (MD) with the linear (L) and non-linear (NL) models across all dimension. The rates are presented at two, four, and six years from baseline visit, and total follow-up, for glaucoma patients in the test dataset. Error bands represent 95% bootstrapped confidence intervals that have been averaged over the five cross-validation data folds. Bold cells represent rates whose confidence intervals do not overlap with MD.

|  | Follow-up Time (years) | | | |
| --- | --- | --- | --- | --- |
| Model | 2 | 4 | 6 | Total |
| *MD* | *0.09 (0.04, 0.14)* | *0.15 (0.09, 0.22)* | *0.22 (0.14, 0.30)* | *0.31 (0.22, 0.41)* |
| L1 | 0.06 (0.02, 0.11) | 0.13 (0.07, 0.20) | 0.18 (0.11, 0.25) | 0.22 (0.15, 0.31) |
| L2 | 0.07 (0.02, 0.13) | 0.10 (0.05, 0.16) | 0.15 (0.08, 0.22) | 0.24 (0.16, 0.33) |
| L3 | 0.12 (0.06, 0.20) | 0.19 (0.12, 0.27) | 0.23 (0.15, 0.32) | 0.31 (0.22, 0.41) |
| L4 | 0.12 (0.06, 0.19) | 0.15 (0.08, 0.22) | 0.14 (0.08, 0.22) | 0.22 (0.14, 0.30) |
| L5 | 0.21 (0.13, 0.29) | 0.24 (0.16, 0.32) | 0.27 (0.19, 0.35) | 0.36 (0.26, 0.45) |
| L6 | 0.11 (0.05, 0.17) | 0.17 (0.09, 0.25) | 0.17 (0.10, 0.24) | 0.21 (0.13, 0.29) |
| L7 | **0.24 (0.16, 0.32)** | 0.27 (0.19, 0.36) | 0.28 (0.19, 0.37) | 0.36 (0.26, 0.45) |
| L8 | **0.25 (0.17, 0.34)** | **0.35 (0.25, 0.44)** | 0.37 (0.27, 0.46) | 0.43 (0.33, 0.52) |
| L9 | 0.10 (0.05, 0.15) | 0.15 (0.08, 0.22) | 0.16 (0.09, 0.24) | 0.23 (0.14, 0.32) |
| L10 | **0.35 (0.26, 0.45)** | **0.42 (0.32, 0.52)** | **0.44 (0.34, 0.54)** | 0.50 (0.41, 0.60) |
| L11 | 0.18 (0.11, 0.25) | 0.21 (0.13, 0.29) | 0.24 (0.16, 0.32) | 0.27 (0.19, 0.35) |
| L12 | 0.08 (0.03, 0.13) | 0.08 (0.04, 0.14) | 0.09 (0.04, 0.15) | 0.12 (0.05, 0.18) |
| L13 | 0.05 (0.01, 0.09) | 0.06 (0.02, 0.10) | 0.07 (0.02, 0.12) | 0.09 (0.04, 0.15) |
| L14 | 0.13 (0.07, 0.20) | 0.17 (0.10, 0.25) | 0.18 (0.11, 0.25) | 0.24 (0.16, 0.33) |
| L15 | 0.03 (0.01, 0.05) | 0.04 (0.01, 0.09) | 0.05 (0.01, 0.10) | 0.09 (0.04, 0.14) |
| NL1 | 0.04 (0.01, 0.08) | 0.10 (0.04, 0.15) | 0.16 (0.09, 0.24) | 0.23 (0.16, 0.32) |
| NL2 | 0.06 (0.02, 0.11) | 0.10 (0.05, 0.17) | 0.13 (0.07, 0.19) | 0.22 (0.13, 0.30) |
| NL3 | 0.11 (0.05, 0.17) | 0.19 (0.11, 0.26) | 0.23 (0.16, 0.32) | 0.33 (0.24, 0.43) |
| NL4 | 0.10 (0.04, 0.17) | 0.16 (0.09, 0.23) | 0.16 (0.09, 0.23) | 0.21 (0.13, 0.29) |
| NL5 | 0.17 (0.10, 0.25) | 0.26 (0.18, 0.35) | 0.29 (0.20, 0.39) | 0.38 (0.29, 0.48) |
| NL6 | 0.09 (0.04, 0.14) | 0.15 (0.08, 0.22) | 0.15 (0.08, 0.23) | 0.21 (0.12, 0.28) |
| NL7 | 0.17 (0.09, 0.23) | 0.29 (0.21, 0.38) | 0.31 (0.22, 0.40) | 0.38 (0.29, 0.48) |
| NL8 | 0.22 (0.14, 0.30) | **0.35 (0.25, 0.44)** | 0.36 (0.27, 0.46) | 0.43 (0.34, 0.53) |
| NL9 | 0.08 (0.03, 0.14) | 0.15 (0.08, 0.21) | 0.17 (0.10, 0.25) | 0.21 (0.13, 0.30) |
| NL10 | **0.28 (0.18, 0.36)** | **0.47 (0.36, 0.57)** | **0.49 (0.40, 0.59)** | **0.55 (0.45, 0.65)** |
| NL11 | 0.13 (0.07, 0.20) | 0.22 (0.14, 0.30) | 0.23 (0.15, 0.32) | 0.28 (0.19, 0.36) |
| NL12 | 0.06 (0.02, 0.11) | 0.07 (0.03, 0.12) | 0.08 (0.04, 0.13) | 0.10 (0.05, 0.16) |
| NL13 | 0.04 (0.01, 0.09) | 0.04 (0.01, 0.09) | 0.07 (0.02, 0.12) | 0.08 (0.03, 0.14) |
| NL14 | 0.11 (0.05, 0.17) | 0.16 (0.09, 0.24) | 0.16 (0.10, 0.23) | 0.23 (0.15, 0.31) |
| NL15 | 0.03 (0.01, 0.05) | 0.04 (0.01, 0.08) | 0.05 (0.02, 0.09) | 0.07 (0.03, 0.13) |

**Supplementary Table S2:** Medians and 95% confidence intervals of mean absolute error (MAE) (in dB) presented over latent dimensions for linear (L) and non-linear (NL) models, along with point-wise (PW) linear regression. Predictions are made using the first **three** visits from **all** patients in the test dataset, with predictions being made to future visits 4-8. Summaries of MAE are presented only for the 52 informative locations (i.e., not the full 12x12 image). P-values correspond to the one-sided Wilcoxon signed rank test, comparing each model to the PW prediction. Cells that are bold correspond to significant p-values based on the Bonferroni corrected type 1 error of 0.00006.

|  | Prediction of Future Visit: | | | | |
| --- | --- | --- | --- | --- | --- |
| Model | 4 | 5 | 6 | 7 | 8 |
| *PW* | *2.99 (1.30, 30.52)* | *4.39 (1.79, 55.88)* | *5.72 (2.06, 72.57)* | *6.79 (2.26, 100.82)* | *8.07 (2.61, 111.00)* |
| L1 | 2.89 (1.04, 18.42) | **3.79 (1.10, 25.36)** | **4.76 (1.18, 28.57)** | **5.49 (1.22, 31.19)** | **6.09 (1.26, 33.90)** |
| L2 | **2.48 (0.99, 13.01)** | **3.20 (1.11, 18.78)** | **3.88 (1.22, 19.78)** | **4.22 (1.22, 21.61)** | **4.89 (1.28, 22.16)** |
| L3 | **2.41 (1.03, 14.55)** | **3.21 (1.14, 18.40)** | **3.80 (1.26, 21.40)** | **4.33 (1.28, 23.54)** | **4.93 (1.31, 24.93)** |
| L4 | **2.35 (1.01, 13.76)** | **3.15 (1.13, 19.58)** | **3.81 (1.24, 20.63)** | **4.29 (1.25, 22.99)** | **4.90 (1.32, 23.68)** |
| L5 | **2.33 (1.04, 17.44)** | **3.11 (1.13, 21.86)** | **3.81 (1.24, 23.92)** | **4.14 (1.27, 25.66)** | **4.74 (1.34, 26.71)** |
| L6 | **2.36 (1.07, 16.56)** | **3.19 (1.16, 21.32)** | **3.87 (1.31, 21.19)** | **4.41 (1.38, 23.86)** | **5.02 (1.43, 25.07)** |
| L7 | **2.37 (1.05, 15.65)** | **3.23 (1.16, 20.60)** | **4.02 (1.27, 21.72)** | **4.56 (1.34, 23.88)** | **5.21 (1.47, 24.98)** |
| L8 | **2.36 (1.00, 15.36)** | **3.30 (1.14, 19.72)** | **4.04 (1.29, 21.65)** | **4.54 (1.31, 23.97)** | **5.14 (1.32, 24.84)** |
| L9 | **2.40 (1.03, 14.50)** | **3.29 (1.22, 20.83)** | **3.97 (1.39, 22.65)** | **4.59 (1.40, 25.13)** | **5.18 (1.45, 26.03)** |
| L10 | **2.45 (1.09, 14.77)** | **3.32 (1.24, 19.68)** | **4.06 (1.40, 21.58)** | **4.54 (1.43, 24.80)** | **5.29 (1.50, 25.41)** |
| L11 | **2.47 (1.05, 14.27)** | **3.31 (1.22, 17.83)** | **4.13 (1.39, 20.67)** | **4.60 (1.47, 24.18)** | **5.29 (1.51, 25.25)** |
| L12 | **2.43 (1.05, 13.72)** | **3.33 (1.21, 18.05)** | **4.23 (1.40, 20.72)** | **4.65 (1.41, 23.82)** | **5.43 (1.54, 24.04)** |
| L13 | **2.44 (1.07, 14.50)** | **3.30 (1.29, 19.58)** | **4.02 (1.45, 20.75)** | **4.57 (1.48, 23.46)** | **5.30 (1.57, 24.54)** |
| L14 | **2.49 (1.08, 15.04)** | **3.35 (1.30, 19.61)** | **4.23 (1.46, 22.20)** | **4.77 (1.47, 24.72)** | **5.47 (1.62, 25.52)** |
| L15 | **2.47 (1.10, 15.20)** | **3.40 (1.31, 22.43)** | **4.19 (1.47, 23.51)** | **4.64 (1.54, 26.71)** | **5.42 (1.67, 27.29)** |
| NL1 | 7.71 (1.23, 36.93) | 13.27 (1.47, 37.45) | 19.07 (1.73, 37.67) | 21.84 (2.09, 37.71) | 22.98 (2.63, 37.92) |
| NL2 | 6.08 (1.20, 35.58) | 13.39 (1.79, 36.35) | 19.59 (2.68, 36.52) | 23.61 (3.32, 36.95) | 25.10 (4.29, 36.89) |
| NL3 | 6.22 (1.27, 33.41) | 13.36 (1.91, 35.21) | 19.12 (3.05, 35.54) | 22.96 (4.25, 35.91) | 25.00 (5.40, 36.23) |
| NL4 | 6.47 (1.25, 32.19) | 14.51 (2.05, 32.92) | 19.85 (3.14, 33.56) | 23.05 (5.11, 34.27) | 24.93 (6.35, 34.18) |
| NL5 | 6.41 (1.29, 34.20) | 15.34 (1.95, 34.36) | 20.51 (2.95, 34.91) | 24.53 (4.37, 35.50) | 26.59 (6.12, 35.04) |
| NL6 | 6.66 (1.27, 35.10) | 16.88 (1.98, 35.04) | 22.53 (2.92, 35.75) | 25.65 (4.33, 36.32) | 27.49 (7.14, 36.17) |
| NL7 | 6.92 (1.25, 36.28) | 17.78 (1.89, 36.92) | 23.06 (3.15, 37.06) | 25.41 (4.20, 37.13) | 27.18 (5.84, 37.42) |
| NL8 | 7.01 (1.26, 36.65) | 17.47 (2.02, 37.22) | 22.88 (3.22, 37.48) | 25.52 (4.67, 37.42) | 27.17 (6.93, 37.65) |
| NL9 | 6.95 (1.29, 36.76) | 17.81 (2.12, 37.08) | 23.02 (3.40, 37.13) | 25.64 (4.54, 37.32) | 27.87 (6.38, 37.48) |
| NL10 | 7.06 (1.39, 35.81) | 16.64 (2.29, 36.38) | 22.34 (3.68, 36.61) | 25.63 (5.19, 36.99) | 27.20 (7.09, 36.87) |
| NL11 | 7.03 (1.37, 36.48) | 17.20 (2.23, 36.80) | 22.86 (3.73, 36.82) | 25.74 (5.13, 37.18) | 27.35 (7.20, 37.60) |
| NL12 | 7.26 (1.36, 36.27) | 17.40 (2.29, 36.40) | 22.32 (3.87, 36.79) | 25.55 (5.63, 36.83) | 27.20 (7.66, 36.77) |
| NL13 | 7.34 (1.40, 36.24) | 17.63 (2.32, 36.38) | 23.26 (4.15, 36.50) | 25.60 (5.72, 36.81) | 27.29 (8.01, 36.84) |
| NL14 | 7.61 (1.40, 35.75) | 17.75 (2.45, 36.39) | 23.17 (4.46, 36.15) | 26.71 (5.94, 36.74) | 28.14 (7.95, 36.87) |
| NL15 | 7.95 (1.40, 36.46) | 18.58 (2.40, 37.12) | 24.68 (4.13, 37.15) | 27.33 (5.99, 37.23) | 28.85 (8.32, 37.35) |

**Supplementary Table S3:** Medians and 95% confidence intervals of mean absolute error (MAE) (in dB) presented over latent dimensions for linear (L) and non-linear (NL) models, along with point-wise (PW) linear regression. Predictions are made using the first **five** visits from **all** patients in the test dataset, with predictions being made to future visits. Summaries of MAE are presented only for the 52 informative locations (i.e., not the full 12x12 image). P-values correspond to the one-sided Wilcoxon signed rank test, comparing each model to the PW prediction. Cells that are bold correspond to significant p-values based on the Bonferroni corrected type 1 error of 0.00006.

|  | Prediction of Future Visit: | | | | |
| --- | --- | --- | --- | --- | --- |
| Model | 6 | 7 | 8 | 9 | 10 |
| *PW* | *2.99 (1.30, 30.52)* | *4.39 (1.79, 55.88)* | *5.72 (2.06, 72.57)* | *6.79 (2.26, 100.82)* | *8.07 (2.61, 111.00)* |
| L1 | 2.15 (1.16, 7.33) | 2.60 (1.28, 8.86) | 3.12 (1.40, 10.27) | **3.50 (1.59, 12.34)** | **3.97 (1.62, 12.67)** |
| L2 | **2.33 (1.00, 12.29)** | **2.62 (1.04, 12.13)** | **2.84 (1.09, 12.90)** | **3.05 (1.10, 14.57)** | **3.19 (1.22, 15.59)** |
| L3 | **1.95 (0.97, 8.21)** | **2.18 (0.99, 8.64)** | **2.42 (1.06, 10.19)** | **2.51 (1.08, 10.69)** | **2.79 (1.07, 10.38)** |
| L4 | **1.90 (1.00, 7.71)** | **2.15 (1.03, 7.65)** | **2.46 (1.08, 9.46)** | **2.61 (1.12, 9.76)** | **2.78 (1.12, 10.12)** |
| L5 | **1.89 (0.98, 7.36)** | **2.16 (1.03, 8.19)** | **2.37 (1.12, 9.95)** | **2.61 (1.11, 10.64)** | **2.91 (1.12, 10.54)** |
| L6 | **1.91 (1.00, 7.35)** | **2.21 (1.01, 7.88)** | **2.43 (1.09, 9.93)** | **2.59 (1.10, 10.26)** | **2.83 (1.13, 11.43)** |
| L7 | **1.99 (1.02, 7.13)** | **2.18 (1.04, 8.15)** | **2.45 (1.12, 10.13)** | **2.60 (1.09, 10.77)** | **2.83 (1.12, 11.85)** |
| L8 | **1.96 (1.00, 7.25)** | **2.15 (1.03, 8.61)** | **2.51 (1.07, 10.49)** | **2.67 (1.15, 12.92)** | **2.96 (1.14, 14.06)** |
| L9 | **1.96 (0.99, 7.40)** | **2.19 (1.02, 8.55)** | **2.49 (1.06, 10.76)** | **2.63 (1.08, 12.75)** | **2.96 (1.12, 14.35)** |
| L10 | **1.95 (1.00, 6.97)** | **2.20 (1.06, 8.07)** | **2.48 (1.14, 10.14)** | **2.61 (1.13, 12.22)** | **2.97 (1.16, 13.78)** |
| L11 | **1.97 (1.00, 6.40)** | **2.20 (1.03, 7.79)** | **2.56 (1.14, 10.03)** | **2.71 (1.15, 11.39)** | **3.03 (1.17, 12.39)** |
| L12 | **1.92 (1.02, 6.55)** | **2.17 (1.04, 7.93)** | **2.58 (1.11, 10.12)** | **2.76 (1.13, 11.04)** | **3.00 (1.21, 11.65)** |
| L13 | **1.92 (0.98, 6.89)** | **2.20 (1.03, 8.00)** | **2.54 (1.12, 9.69)** | **2.74 (1.14, 10.51)** | **3.05 (1.17, 11.82)** |
| L14 | **1.91 (0.98, 6.53)** | **2.18 (1.03, 7.91)** | **2.54 (1.11, 10.13)** | **2.71 (1.16, 10.23)** | **3.01 (1.20, 11.23)** |
| L15 | **1.96 (0.98, 6.67)** | **2.21 (1.05, 7.79)** | **2.58 (1.11, 9.39)** | **2.80 (1.11, 10.40)** | **3.14 (1.24, 11.19)** |
| NL1 | 1.96 (1.01, 6.68) | 2.26 (1.06, 7.73) | 2.60 (1.13, 9.86) | 2.75 (1.18, 10.47) | 3.04 (1.25, 11.32) |
| NL2 | 3.23 (1.06, 22.15) | 4.73 (1.16, 30.56) | 6.47 (1.27, 34.64) | 8.56 (1.24, 37.15) | 10.43 (1.34, 37.01) |
| NL3 | 2.65 (1.06, 15.09) | 3.96 (1.12, 24.25) | 5.48 (1.29, 27.38) | 7.04 (1.36, 28.80) | 9.05 (1.47, 31.51) |
| NL4 | 2.61 (1.06, 13.87) | 3.92 (1.20, 21.71) | 5.59 (1.33, 25.59) | 7.08 (1.55, 28.17) | 9.22 (1.85, 29.88) |
| NL5 | 2.70 (1.06, 15.21) | 3.99 (1.17, 21.54) | 6.05 (1.37, 25.97) | 8.04 (1.45, 27.59) | 10.34 (1.71, 28.39) |
| NL6 | 2.64 (1.05, 20.31) | 4.02 (1.15, 23.95) | 5.81 (1.42, 27.05) | 8.00 (1.59, 29.63) | 10.28 (1.72, 30.93) |
| NL7 | 2.70 (1.10, 18.67) | 4.25 (1.26, 24.75) | 6.26 (1.40, 28.44) | 8.77 (1.71, 29.40) | 11.44 (1.80, 31.76) |
| NL8 | 2.72 (1.11, 22.14) | 4.22 (1.22, 28.90) | 6.49 (1.49, 29.05) | 8.70 (1.75, 32.36) | 11.42 (1.90, 34.11) |
| NL9 | 2.73 (1.07, 22.99) | 4.28 (1.23, 29.14) | 6.35 (1.44, 31.44) | 8.70 (1.52, 33.40) | 11.05 (1.88, 35.00) |
| NL10 | 2.75 (1.09, 22.10) | 4.24 (1.23, 29.88) | 6.30 (1.50, 30.41) | 8.28 (1.85, 33.44) | 10.75 (1.92, 35.52) |
| NL11 | 2.79 (1.14, 22.21) | 4.42 (1.33, 28.06) | 6.70 (1.64, 30.83) | 8.60 (1.90, 32.28) | 10.91 (2.20, 33.22) |
| NL12 | 2.79 (1.09, 20.41) | 4.41 (1.33, 27.89) | 6.72 (1.63, 30.27) | 8.92 (1.97, 32.97) | 10.94 (2.35, 33.90) |
| NL13 | 2.80 (1.11, 21.42) | 4.42 (1.36, 27.98) | 6.79 (1.60, 30.43) | 9.04 (2.06, 32.50) | 11.08 (2.37, 33.80) |
| NL14 | 2.80 (1.10, 21.06) | 4.32 (1.36, 28.31) | 6.85 (1.61, 30.53) | 9.10 (2.05, 31.22) | 11.48 (2.42, 33.07) |
| NL15 | 2.87 (1.14, 21.35) | 4.50 (1.33, 29.08) | 6.76 (1.63, 30.81) | 9.21 (2.04, 32.21) | 11.48 (2.64, 33.65) |

**Supplementary Table S4:** Medians and 95% confidence intervals of mean absolute error (MAE) (in dB) presented over latent dimensions for linear (L) and non-linear (NL) models, along with point-wise (PW) linear regression. Predictions are made using the first **seven** visits from **all** patients in the test dataset, with predictions being made to future visits. Summaries of MAE are presented only for the 52 informative locations (i.e., not the full 12x12 image). P-values correspond to the one-sided Wilcoxon signed rank test, comparing each model to the PW prediction. Cells that are bold correspond to significant p-values based on the Bonferroni corrected type 1 error of 0.00006.

|  | Prediction of Future Visit: | | | | |
| --- | --- | --- | --- | --- | --- |
| Model | 8 | 9 | 10 | 11 | 12 |
| *PW* | *1.99 (1.08, 5.41)* | *2.19 (1.16, 6.06)* | *2.43 (1.23, 7.39)* | *2.79 (1.32, 7.97)* | *3.02 (1.40, 9.08)* |
| L1 | 2.30 (1.09, 9.86) | 2.47 (1.08, 10.10) | 2.61 (1.03, 11.71) | 2.68 (1.12, 12.38) | 2.81 (1.06, 13.49) |
| L2 | 1.88 (0.99, 7.40) | 1.98 (1.00, 7.65) | **2.09 (0.98, 8.19)** | **2.26 (1.07, 8.35)** | **2.37 (1.08, 8.65)** |
| L3 | 1.89 (0.97, 6.87) | **1.98 (1.02, 7.09)** | **2.11 (1.02, 7.47)** | **2.23 (1.07, 8.07)** | **2.36 (1.11, 8.39)** |
| L4 | 1.89 (0.95, 6.75) | **1.95 (1.03, 7.09)** | **2.12 (1.03, 7.47)** | **2.29 (1.05, 7.95)** | **2.40 (1.10, 8.80)** |
| L5 | 1.91 (1.00, 6.24) | **1.96 (1.04, 6.71)** | **2.10 (1.01, 7.18)** | **2.23 (1.06, 8.19)** | **2.43 (1.08, 8.42)** |
| L6 | 1.90 (1.03, 6.23) | **2.00 (1.06, 6.40)** | **2.13 (1.08, 7.78)** | **2.30 (1.09, 7.66)** | **2.40 (1.11, 8.15)** |
| L7 | 1.85 (0.98, 6.19) | **1.93 (1.05, 6.72)** | **2.10 (1.02, 8.21)** | **2.29 (1.06, 8.38)** | **2.43 (1.11, 9.17)** |
| L8 | 1.86 (1.00, 6.38) | **1.94 (1.01, 6.64)** | **2.06 (1.06, 8.08)** | **2.27 (1.06, 8.44)** | **2.39 (1.11, 9.35)** |
| L9 | 1.87 (1.02, 6.00) | **1.98 (1.01, 6.47)** | **2.12 (1.08, 7.53)** | **2.24 (1.11, 8.05)** | **2.42 (1.14, 8.62)** |
| L10 | 1.89 (1.03, 5.53) | **1.97 (1.07, 6.32)** | **2.09 (1.07, 7.41)** | **2.31 (1.06, 7.74)** | **2.41 (1.12, 8.45)** |
| L11 | **1.89 (1.01, 5.80)** | **1.94 (1.02, 6.13)** | **2.16 (1.05, 7.45)** | **2.31 (1.08, 7.90)** | **2.48 (1.13, 8.31)** |
| L12 | **1.86 (0.98, 5.63)** | **1.97 (1.04, 6.19)** | **2.13 (1.06, 6.98)** | **2.32 (1.08, 7.29)** | **2.48 (1.12, 8.18)** |
| L13 | **1.84 (0.98, 5.47)** | **1.93 (1.00, 5.94)** | **2.09 (1.05, 7.32)** | **2.24 (1.06, 7.36)** | **2.46 (1.09, 8.01)** |
| L14 | **1.89 (0.98, 5.49)** | **2.01 (1.02, 5.88)** | **2.16 (1.02, 6.81)** | **2.36 (1.06, 7.48)** | **2.51 (1.17, 8.02)** |
| L15 | **1.90 (0.99, 5.26)** | **1.97 (1.04, 5.84)** | **2.15 (1.05, 7.12)** | **2.34 (1.11, 7.51)** | **2.49 (1.17, 8.11)** |
| NL1 | 2.68 (1.06, 12.24) | 3.64 (1.18, 16.64) | 4.66 (1.24, 28.39) | 5.71 (1.20, 32.94) | 6.53 (1.31, 34.78) |
| NL2 | 2.29 (1.01, 7.90) | 3.00 (1.11, 10.71) | 3.54 (1.16, 13.72) | 4.46 (1.23, 17.56) | 5.35 (1.34, 21.34) |
| NL3 | 2.25 (1.00, 7.68) | 2.98 (1.15, 10.45) | 3.72 (1.21, 13.30) | 4.40 (1.25, 18.64) | 5.29 (1.38, 22.67) |
| NL4 | 2.29 (1.01, 8.01) | 3.04 (1.12, 10.01) | 3.87 (1.25, 13.95) | 4.70 (1.31, 18.84) | 5.87 (1.42, 22.89) |
| NL5 | 2.34 (1.02, 7.86) | 3.00 (1.09, 11.67) | 3.87 (1.21, 15.48) | 4.54 (1.35, 21.28) | 5.54 (1.46, 24.41) |
| NL6 | 2.35 (1.08, 8.55) | 3.07 (1.17, 13.58) | 3.98 (1.22, 17.96) | 4.78 (1.34, 24.21) | 5.99 (1.39, 26.20) |
| NL7 | 2.31 (1.02, 9.17) | 3.04 (1.17, 13.34) | 4.00 (1.26, 19.85) | 4.98 (1.37, 27.22) | 6.22 (1.48, 29.19) |
| NL8 | 2.30 (1.00, 9.00) | 3.03 (1.14, 14.44) | 4.02 (1.18, 20.02) | 5.01 (1.33, 26.74) | 5.92 (1.45, 28.85) |
| NL9 | 2.30 (1.05, 9.02) | 3.07 (1.19, 12.93) | 4.06 (1.29, 18.90) | 4.83 (1.37, 25.57) | 5.97 (1.48, 28.58) |
| NL10 | 2.34 (1.04, 8.49) | 3.14 (1.21, 12.04) | 4.15 (1.36, 16.59) | 4.97 (1.57, 22.99) | 6.15 (1.69, 26.27) |
| NL11 | 2.37 (1.05, 8.73) | 3.19 (1.18, 12.73) | 4.18 (1.37, 17.72) | 5.11 (1.54, 25.41) | 6.31 (1.73, 27.93) |
| NL12 | 2.38 (1.03, 8.75) | 3.18 (1.21, 12.00) | 4.15 (1.35, 18.72) | 5.21 (1.59, 23.92) | 6.24 (1.70, 27.27) |
| NL13 | 2.34 (1.06, 9.35) | 3.16 (1.19, 12.52) | 4.09 (1.37, 18.32) | 5.20 (1.59, 23.82) | 6.30 (1.74, 26.68) |
| NL14 | 2.38 (1.08, 9.12) | 3.25 (1.24, 12.64) | 4.22 (1.30, 16.94) | 5.20 (1.60, 23.26) | 6.34 (1.69, 27.05) |
| NL15 | 2.39 (1.09, 9.43) | 3.24 (1.28, 12.51) | 4.20 (1.46, 17.86) | 5.43 (1.64, 25.10) | 6.56 (1.79, 28.68) |

**Supplementary Table S5:** Medians and 95% confidence intervals of mean absolute error (MAE) (in dB) presented over latent dimensions for linear (L) and non-linear (NL) models, along with point-wise (PW) linear regression. Predictions are made using the first **three** visits from **glaucoma** patients in the test dataset, with predictions being made to future visits. Summaries of MAE are presented only for the 52 informative locations (i.e., not the full 12x12 image). P-values correspond to the one-sided Wilcoxon signed rank test, comparing each model to the PW prediction. Cells that are bold correspond to significant p-values based on the Bonferroni corrected type 1 error of 0.00006.

|  | Prediction of Future Visit: | | | | |
| --- | --- | --- | --- | --- | --- |
| Model | 4 | 5 | 6 | 7 | 8 |
| *PW* | *4.18 (1.82, 20.69)* | *6.07 (2.62, 36.69)* | *7.95 (3.01, 56.76)* | *9.20 (3.56, 72.54)* | *10.79 (4.00, 89.76)* |
| L1 | 5.39 ( 1.63, 17.65) | 6.07 ( 1.73, 21.35) | **6.42 ( 1.66, 25.04)** | **6.92 ( 1.82, 25.56)** | **7.04 ( 1.80, 26.81)** |
| L2 | **4.23 ( 1.70, 12.20)** | **4.78 ( 1.77, 15.31)** | **5.28 ( 1.64, 19.26)** | **5.70 ( 1.69, 20.94)** | **6.23 ( 1.78, 21.11)** |
| L3 | **4.19 ( 1.79, 12.39)** | **4.73 ( 1.81, 15.19)** | **5.34 ( 1.98, 19.13)** | **5.77 ( 2.04, 20.56)** | **6.25 ( 2.10, 21.74)** |
| L4 | **4.06 ( 1.72, 12.60)** | **4.81 ( 1.82, 15.37)** | **5.49 ( 1.78, 19.80)** | **5.65 ( 2.14, 20.88)** | **6.17 ( 2.25, 21.33)** |
| L5 | **4.00 ( 1.67, 13.11)** | **4.83 ( 1.84, 16.44)** | **5.60 ( 2.06, 18.80)** | **6.00 ( 2.21, 19.97)** | **6.41 ( 2.50, 22.26)** |
| L6 | **4.07 ( 1.63, 14.16)** | **5.01 ( 1.76, 17.85)** | **5.77 ( 1.92, 20.53)** | **6.33 ( 2.23, 20.89)** | **6.99 ( 2.49, 24.19)** |
| L7 | **4.02 ( 1.63, 16.96)** | **5.03 ( 1.78, 20.60)** | **5.90 ( 1.78, 23.78)** | **6.62 ( 2.14, 24.71)** | **7.84 ( 2.20, 25.65)** |
| L8 | **3.94 ( 1.61, 15.08)** | **5.13 ( 1.79, 20.53)** | **6.10 ( 1.96, 24.18)** | **6.87 ( 2.09, 24.59)** | **7.66 ( 2.32, 26.68)** |
| L9 | **4.05 ( 1.70, 14.12)** | **4.98 ( 1.92, 20.54)** | **5.88 ( 1.97, 22.72)** | **6.73 ( 2.10, 24.83)** | **7.51 ( 2.34, 25.84)** |
| L10 | **3.95 ( 1.63, 13.11)** | **4.95 ( 1.91, 17.02)** | **5.75 ( 2.12, 21.56)** | **6.57 ( 2.15, 23.20)** | **7.43 ( 2.46, 24.69)** |
| L11 | **3.97 ( 1.74, 13.31)** | **5.01 ( 1.91, 16.96)** | **6.00 ( 1.91, 20.59)** | **6.59 ( 2.34, 23.19)** | **7.65 ( 2.76, 25.17)** |
| L12 | **3.86 ( 1.65, 12.45)** | **5.04 ( 1.91, 15.79)** | **5.90 ( 2.08, 20.44)** | **6.88 ( 2.21, 22.60)** | **7.66 ( 2.37, 23.39)** |
| L13 | **3.93 ( 1.73, 12.63)** | **5.03 ( 1.88, 17.45)** | **6.06 ( 2.14, 19.54)** | **7.01 ( 2.36, 21.65)** | **7.72 ( 2.77, 23.81)** |
| L14 | **3.88 ( 1.70, 12.34)** | **5.09 ( 2.01, 17.28)** | **6.15 ( 2.18, 20.82)** | **6.71 ( 2.54, 21.55)** | **7.66 ( 2.91, 23.56)** |
| L15 | **3.91 ( 1.69, 13.05)** | **5.19 ( 2.02, 20.48)** | **6.10 ( 2.31, 21.85)** | **7.17 ( 2.44, 23.39)** | **7.86 ( 2.87, 26.83)** |
| NL1 | 8.14 ( 1.64, 32.37) | 13.12 ( 2.18, 35.02) | 17.89 ( 2.45, 36.68) | 21.54 ( 3.03, 36.43) | 22.93 ( 4.49, 36.09) |
| NL2 | 7.48 ( 1.95, 31.95) | 14.03 ( 2.79, 33.29) | 19.60 ( 3.77, 34.86) | 22.90 ( 4.15, 35.25) | 24.74 ( 6.10, 35.77) |
| NL3 | 7.90 ( 2.04, 32.00) | 15.15 ( 2.97, 33.59) | 20.09 ( 3.85, 33.98) | 22.83 ( 4.91, 34.90) | 25.49 ( 7.19, 34.66) |
| NL4 | 8.63 ( 2.21, 31.75) | 15.87 ( 3.06, 32.31) | 21.59 ( 3.52, 33.19) | 24.06 ( 5.36, 33.77) | 25.63 ( 7.74, 34.50) |
| NL5 | 8.68 ( 2.27, 31.67) | 17.39 ( 3.22, 33.13) | 22.10 ( 5.18, 33.34) | 25.20 ( 7.01, 33.79) | 26.81 ( 8.98, 34.12) |
| NL6 | 9.41 ( 2.28, 32.57) | 18.92 ( 3.61, 33.74) | 22.90 ( 5.03, 34.66) | 24.84 ( 6.71, 34.56) | 26.91 ( 9.04, 35.23) |
| NL7 | 9.11 ( 2.34, 33.47) | 19.19 ( 3.57, 33.38) | 23.30 ( 5.32, 34.79) | 25.05 ( 7.05, 34.94) | 26.84 ( 9.34, 35.65) |
| NL8 | 9.04 ( 2.21, 34.13) | 19.21 ( 3.57, 34.90) | 23.24 ( 5.35, 35.96) | 24.99 ( 6.48, 36.53) | 26.70 ( 9.23, 36.38) |
| NL9 | 8.67 ( 2.27, 33.30) | 19.37 ( 3.71, 34.62) | 22.81 ( 5.10, 35.29) | 24.85 ( 6.27, 35.69) | 26.77 ( 9.80, 35.91) |
| NL10 | 9.28 ( 2.31, 33.44) | 18.42 ( 3.86, 34.35) | 22.33 ( 5.53, 35.26) | 24.76 ( 6.55, 34.96) | 27.00 ( 9.68, 36.17) |
| NL11 | 9.28 ( 2.48, 33.86) | 18.40 ( 3.97, 34.27) | 22.34 ( 5.73, 35.31) | 24.94 ( 6.49, 35.77) | 26.63 (11.66, 36.08) |
| NL12 | 9.18 ( 2.30, 32.98) | 18.53 ( 3.98, 33.92) | 22.02 ( 5.72, 34.31) | 25.22 ( 6.01, 34.83) | 26.60 (10.95, 35.58) |
| NL13 | 9.71 ( 2.49, 33.35) | 19.02 ( 4.16, 34.22) | 23.33 ( 5.61, 35.15) | 25.46 ( 7.06, 35.00) | 26.89 (11.26, 35.64) |
| NL14 | 9.72 ( 2.56, 33.85) | 18.11 ( 4.18, 34.29) | 22.93 ( 6.26, 34.87) | 25.56 ( 7.51, 34.67) | 27.60 (11.80, 35.22) |
| NL15 | 10.24 ( 2.52, 33.73) | 20.26 ( 4.17, 34.14) | 24.23 ( 6.02, 35.26) | 25.73 ( 7.21, 35.36) | 27.83 (10.24, 35.80) |

**Supplementary Table S6:** Medians and 95% confidence intervals of mean absolute error (MAE) (in dB) presented over latent dimensions for linear (L) and non-linear (NL) models, along with point-wise (PW) linear regression. Predictions are made using the first **five** visits from **glaucoma** patients in the test dataset, with predictions being made to future visits. Summaries of MAE are presented only for the 52 informative locations (i.e., not the full 12x12 image). P-values correspond to the one-sided Wilcoxon signed rank test, comparing each model to the PW prediction. Cells that are bold correspond to significant p-values based on the Bonferroni corrected type 1 error of 0.00006.

|  | Prediction of Future Visit: | | | | |
| --- | --- | --- | --- | --- | --- |
| Model | 6 | 7 | 8 | 9 | 10 |
| *PW* | *3.29 (1.54, 8.23)* | *4.08 (1.81, 10.32)* | *4.68 (2.24, 12.46)* | *5.33 (2.51, 13.88)* | *6.41 (2.73, 16.70)* |
| L1 | 4.89 ( 1.40, 14.28) | 5.11 ( 1.45, 13.61) | 5.04 ( 1.68, 14.79) | 5.14 ( 1.55, 16.90) | 5.44 ( 1.81, 16.36) |
| L2 | 3.97 ( 1.43, 9.51) | 4.19 ( 1.45, 9.90) | **4.37 ( 1.92, 11.94)** | **4.50 ( 1.68, 11.83)** | **4.92 ( 1.89, 13.06)** |
| L3 | 3.87 ( 1.36, 8.29) | 4.20 ( 1.42, 8.19) | **4.08 ( 1.93, 11.73)** | **4.42 ( 1.85, 11.93)** | **4.89 ( 1.88, 12.17)** |
| L4 | 3.74 ( 1.35, 8.18) | 3.98 ( 1.48, 10.04) | **4.23 ( 1.91, 12.72)** | **4.61 ( 1.89, 13.65)** | **4.94 ( 1.94, 14.92)** |
| L5 | 3.65 ( 1.40, 8.09) | 3.96 ( 1.49, 10.79) | **4.29 ( 1.94, 14.04)** | **4.51 ( 1.77, 14.41)** | **4.93 ( 1.85, 14.77)** |
| L6 | 3.55 ( 1.41, 8.67) | **4.00 ( 1.55, 10.00)** | **4.21 ( 1.83, 12.06)** | **4.63 ( 1.86, 13.99)** | **5.02 ( 1.95, 14.22)** |
| L7 | 3.62 ( 1.36, 8.47) | **3.90 ( 1.56, 11.54)** | **4.35 ( 1.90, 14.93)** | **4.65 ( 2.10, 17.23)** | **5.17 ( 2.00, 16.89)** |
| L8 | 3.58 ( 1.40, 9.69) | 3.91 ( 1.43, 12.28) | **4.19 ( 1.95, 16.65)** | **4.48 ( 1.94, 18.13)** | **4.98 ( 1.98, 19.49)** |
| L9 | 3.55 ( 1.36, 7.95) | **3.92 ( 1.44, 10.40)** | **4.34 ( 1.93, 14.27)** | **4.61 ( 1.92, 15.80)** | **5.03 ( 1.98, 16.94)** |
| L10 | 3.50 ( 1.40, 8.29) | **3.81 ( 1.50, 10.33)** | **4.24 ( 1.97, 14.37)** | **4.63 ( 1.88, 15.33)** | **5.00 ( 1.80, 16.52)** |
| L11 | 3.46 ( 1.33, 8.08) | **3.84 ( 1.45, 9.89)** | **4.23 ( 2.04, 12.12)** | **4.58 ( 1.87, 12.99)** | **4.97 ( 2.03, 14.05)** |
| L12 | 3.40 ( 1.35, 7.69) | **3.75 ( 1.49, 8.95)** | **4.16 ( 1.92, 12.33)** | **4.59 ( 1.85, 13.20)** | **4.97 ( 2.05, 13.35)** |
| L13 | 3.39 ( 1.39, 8.45) | **3.78 ( 1.51, 9.53)** | **4.11 ( 1.94, 12.67)** | **4.52 ( 1.77, 13.97)** | **4.90 ( 1.95, 15.00)** |
| L14 | 3.36 ( 1.42, 7.71) | **3.79 ( 1.51, 8.99)** | **4.19 ( 2.04, 10.02)** | **4.56 ( 1.99, 11.88)** | **4.96 ( 2.19, 12.84)** |
| L15 | 3.44 ( 1.50, 7.80) | **3.79 ( 1.58, 9.43)** | **4.20 ( 1.98, 11.34)** | **4.55 ( 1.99, 13.79)** | **4.98 ( 2.03, 13.14)** |
| NL1 | 5.70 ( 1.68, 16.62) | 6.36 ( 1.85, 20.81) | 7.62 ( 2.02, 30.75) | 9.29 ( 2.38, 33.18) | 10.71 ( 2.65, 33.78) |
| NL2 | 4.54 ( 1.46, 13.30) | 6.13 ( 1.90, 19.30) | 7.59 ( 2.39, 25.64) | 9.80 ( 3.03, 27.79) | 12.02 ( 4.26, 31.13) |
| NL3 | 4.60 ( 1.44, 13.53) | 5.96 ( 1.78, 19.81) | 8.27 ( 2.30, 24.69) | 9.76 ( 3.14, 26.52) | 13.46 ( 4.01, 29.65) |
| NL4 | 4.66 ( 1.46, 15.19) | 6.29 ( 1.92, 20.98) | 8.87 ( 2.68, 25.82) | 11.22 ( 3.28, 27.22) | 14.51 ( 4.28, 28.37) |
| NL5 | 4.73 ( 1.49, 21.01) | 6.40 ( 2.04, 23.39) | 9.10 ( 2.47, 26.92) | 11.46 ( 3.11, 28.94) | 14.71 ( 4.53, 30.89) |
| NL6 | 4.80 ( 1.68, 18.42) | 6.73 ( 2.20, 23.71) | 10.04 ( 3.05, 27.29) | 13.00 ( 3.75, 28.41) | 16.43 ( 5.21, 31.62) |
| NL7 | 4.58 ( 1.59, 21.64) | 6.91 ( 2.03, 24.45) | 10.41 ( 2.98, 27.18) | 12.97 ( 3.65, 29.22) | 15.69 ( 4.50, 30.96) |
| NL8 | 4.46 ( 1.46, 21.93) | 6.78 ( 1.96, 24.86) | 9.73 ( 2.64, 29.62) | 12.65 ( 3.50, 30.93) | 16.08 ( 4.70, 32.79) |
| NL9 | 4.49 ( 1.52, 20.50) | 6.77 ( 2.20, 25.94) | 9.48 ( 3.11, 27.81) | 11.65 ( 3.72, 30.41) | 15.58 ( 5.10, 32.71) |
| NL10 | 4.55 ( 1.47, 18.53) | 6.87 ( 2.14, 22.41) | 9.15 ( 3.05, 27.39) | 11.67 ( 3.91, 30.15) | 14.93 ( 5.69, 31.38) |
| NL11 | 4.45 ( 1.43, 16.72) | 6.87 ( 2.17, 24.01) | 9.62 ( 3.18, 28.55) | 12.25 ( 4.05, 29.88) | 15.59 ( 5.52, 32.25) |
| NL12 | 4.57 ( 1.59, 16.92) | 7.01 ( 2.18, 23.75) | 10.02 ( 3.42, 28.36) | 12.37 ( 4.02, 28.99) | 14.83 ( 5.66, 31.53) |
| NL13 | 4.50 ( 1.55, 18.50) | 7.07 ( 2.11, 24.25) | 9.90 ( 3.53, 27.41) | 11.79 ( 4.34, 29.86) | 15.43 ( 6.07, 31.99) |
| NL14 | 4.45 ( 1.61, 16.91) | 7.24 ( 2.39, 22.43) | 10.40 ( 3.43, 27.10) | 12.17 ( 4.42, 28.95) | 15.49 ( 5.90, 31.05) |
| NL15 | 4.52 ( 1.65, 19.51) | 7.14 ( 2.25, 24.31) | 10.31 ( 3.60, 29.21) | 12.75 ( 4.36, 29.15) | 16.57 ( 5.81, 30.67) |

**Supplementary Table S7:** Medians and 95% confidence intervals of mean absolute error (MAE) (in dB) presented over latent dimensions for linear (L) and non-linear (NL) models, along with point-wise (PW) linear regression. Predictions are made using the first **seven** visits from **glaucoma** patients in the test dataset, with predictions being made to future visits. Summaries of MAE are presented only for the 52 informative locations (i.e., not the full 12x12 image). P-values correspond to the one-sided Wilcoxon signed rank test, comparing each model to the PW prediction. Cells that are bold correspond to significant p-values based on the Bonferroni corrected type 1 error of 0.00006.

|  | Prediction of Future Visit: | | | | | | | | |  |
| --- | --- | --- | --- | --- | --- | --- | --- | --- | --- | --- |
| Model | 8 | 9 | | 10 | | 11 | | 12 | |  |
| *PW* | *2.88 (1.55, 5.98)* | | *3.25 (1.71, 8.01)* | | *3.71 (1.90, 8.63)* | | *4.09 (2.06, 10.35)* | | *4.47 (2.04, 11.58)* | |
| L1 | 4.87 ( 1.61, 12.93) | | 4.86 ( 1.54, 14.46) | | 5.01 ( 1.62, 14.79) | | 5.20 ( 1.59, 14.20) | | 4.89 ( 1.51, 16.45) | |
| L2 | 3.77 ( 1.50, 8.49) | | 3.95 ( 1.58, 8.70) | | 4.15 ( 1.62, 9.15) | | 4.24 ( 1.51, 9.26) | | **4.04 ( 1.47, 10.68)** | |
| L3 | 3.48 ( 1.51, 8.01) | | 3.61 ( 1.55, 8.75) | | 3.94 ( 1.61, 8.52) | | 4.25 ( 1.49, 8.76) | | **3.97 ( 1.44, 9.42)** | |
| L4 | 3.34 ( 1.58, 7.24) | | 3.54 ( 1.42, 8.57) | | 3.77 ( 1.61, 8.88) | | 4.18 ( 1.51, 9.24) | | **3.98 ( 1.44, 10.86)** | |
| L5 | 3.42 ( 1.52, 6.75) | | 3.66 ( 1.54, 7.53) | | 3.84 ( 1.62, 8.36) | | 4.06 ( 1.61, 9.74) | | **3.91 ( 1.53, 11.87)** | |
| L6 | 3.40 ( 1.46, 6.74) | | 3.59 ( 1.56, 7.98) | | 3.82 ( 1.57, 8.17) | | 4.06 ( 1.76, 9.98) | | **3.95 ( 1.61, 10.26)** | |
| L7 | 3.32 ( 1.55, 7.10) | | 3.55 ( 1.54, 8.32) | | 3.83 ( 1.64, 9.24) | | 4.08 ( 1.60, 11.16) | | **4.14 ( 1.50, 12.66)** | |
| L8 | 3.41 ( 1.52, 6.92) | | 3.56 ( 1.61, 7.85) | | 3.71 ( 1.70, 9.40) | | 3.98 ( 1.63, 10.44) | | **4.11 ( 1.65, 11.94)** | |
| L9 | 3.31 ( 1.56, 6.75) | | 3.55 ( 1.56, 8.17) | | 3.72 ( 1.69, 9.30) | | 4.00 ( 1.63, 10.31) | | **4.14 ( 1.53, 11.09)** | |
| L10 | 3.23 ( 1.46, 6.57) | | 3.59 ( 1.56, 8.04) | | 3.64 ( 1.66, 8.91) | | **3.94 ( 1.64, 10.68)** | | **4.02 ( 1.61, 11.75)** | |
| L11 | 3.23 ( 1.47, 6.38) | | 3.54 ( 1.56, 7.90) | | 3.79 ( 1.66, 8.58) | | **3.96 ( 1.63, 9.56)** | | **4.07 ( 1.58, 9.92)** | |
| L12 | 3.23 ( 1.48, 6.49) | | 3.46 ( 1.56, 7.99) | | 3.65 ( 1.75, 8.51) | | **3.85 ( 1.67, 10.13)** | | **3.97 ( 1.55, 10.24)** | |
| L13 | 3.16 ( 1.51, 6.23) | | 3.42 ( 1.59, 8.12) | | 3.68 ( 1.65, 8.67) | | **4.00 ( 1.70, 9.57)** | | **3.97 ( 1.59, 9.71)** | |
| L14 | 3.12 ( 1.48, 6.21) | | 3.38 ( 1.56, 7.59) | | **3.67 ( 1.72, 8.36)** | | **3.91 ( 1.77, 8.36)** | | **4.02 ( 1.72, 9.73)** | |
| L15 | 3.16 ( 1.53, 6.29) | | 3.36 ( 1.59, 7.07) | | 3.66 ( 1.77, 8.82) | | **3.88 ( 1.80, 9.27)** | | **3.96 ( 1.56, 10.18)** | |
| NL1 | 4.90 ( 1.71, 16.01) | | 5.95 ( 1.55, 17.46) | | 6.84 ( 1.69, 23.97) | | 7.75 ( 1.71, 29.22) | | 8.20 ( 1.61, 33.41) | |
| NL2 | 4.13 ( 1.64, 9.74) | | 4.70 ( 1.60, 12.58) | | 5.39 ( 1.56, 15.43) | | 6.07 ( 1.88, 19.90) | | 7.11 ( 1.95, 22.39) | |
| NL3 | 3.97 ( 1.70, 8.96) | | 4.71 ( 1.82, 12.29) | | 5.49 ( 1.65, 16.99) | | 6.29 ( 2.00, 20.19) | | 7.63 ( 2.21, 24.71) | |
| NL4 | 3.80 ( 1.77, 9.81) | | 4.57 ( 1.82, 11.60) | | 5.71 ( 1.94, 19.12) | | 6.90 ( 2.08, 20.55) | | 8.34 ( 2.69, 23.56) | |
| NL5 | 3.79 ( 1.67, 10.24) | | 4.59 ( 1.92, 14.21) | | 5.86 ( 1.92, 21.04) | | 6.94 ( 2.45, 24.19) | | 8.76 ( 2.73, 28.57) | |
| NL6 | 3.83 ( 1.66, 11.23) | | 4.75 ( 1.88, 14.40) | | 6.47 ( 2.00, 22.12) | | 8.05 ( 2.46, 26.42) | | 9.76 ( 2.98, 27.73) | |
| NL7 | 3.77 ( 1.56, 12.58) | | 4.56 ( 1.96, 14.65) | | 5.99 ( 1.83, 23.93) | | 7.83 ( 2.65, 27.69) | | 10.51 ( 2.84, 28.96) | |
| NL8 | 3.79 ( 1.71, 13.48) | | 4.74 ( 1.81, 16.88) | | 6.39 ( 2.13, 25.78) | | 7.61 ( 2.77, 28.69) | | 9.85 ( 2.96, 29.59) | |
| NL9 | 3.80 ( 1.72, 13.65) | | 4.63 ( 1.95, 17.57) | | 5.91 ( 1.92, 22.36) | | 7.43 ( 2.51, 26.24) | | 9.24 ( 2.93, 28.53) | |
| NL10 | 3.77 ( 1.62, 11.76) | | 4.83 ( 2.05, 14.16) | | 5.89 ( 2.14, 20.61) | | 7.49 ( 2.77, 26.07) | | 9.39 ( 2.96, 27.69) | |
| NL11 | 3.70 ( 1.73, 12.87) | | 4.82 ( 1.95, 16.17) | | 6.19 ( 2.29, 22.99) | | 7.92 ( 2.63, 26.49) | | 9.98 ( 2.89, 29.44) | |
| NL12 | 3.69 ( 1.68, 11.97) | | 4.68 ( 2.12, 14.59) | | 6.11 ( 2.33, 24.04) | | 7.42 ( 2.84, 25.61) | | 9.95 ( 3.01, 28.47) | |
| NL13 | 3.70 ( 1.72, 14.06) | | 4.87 ( 2.11, 13.64) | | 5.97 ( 2.35, 22.15) | | 7.82 ( 3.01, 25.30) | | 10.19 ( 3.29, 27.29) | |
| NL14 | 3.72 ( 1.77, 12.36) | | 4.94 ( 2.11, 13.53) | | 6.00 ( 2.43, 20.97) | | 8.14 ( 3.05, 25.00) | | 10.20 ( 3.19, 27.72) | |
| NL15 | 3.68 ( 1.76, 11.59) | | 4.89 ( 2.18, 14.25) | | 6.21 ( 2.38, 22.14) | | 7.84 ( 2.94, 25.52) | | 10.09 ( 3.18, 29.31) | |

End of Supplementary Information.
